# MiR-199a Inhibits Tumor Growth and Attenuates Chemoresistance by Targeting K-RAS via AKT and ERK Signalings

**DOI:** 10.3389/fonc.2019.01071

**Published:** 2019-10-15

**Authors:** Wei Li, Lin Wang, Xiang-Bo Ji, Li-Hong Wang, Xin Ge, Wei-Tao Liu, Ling Chen, Zhong Zheng, Zhu-Mei Shi, Ling-Zhi Liu, Marie C. Lin, Jie-Yu Chen, Bing-Hua Jiang

**Affiliations:** ^1^Department of Pathology, Affiliated Drum Tower Hospital of Nanjing University Medical School, Nanjing, China; ^2^Institute of Medical and Pharmaceutical Sciences, The Academy of Medical Sciences, Zhengzhou University, Zhengzhou, China; ^3^Key Laboratory of Human Functional Genomics of Jiangsu Province, Department of Pathology, Nanjing Medical University, Nanjing, China; ^4^Department of Pathology, Carver College of Medicine, The University of Iowa, Iowa, IA, United States

**Keywords:** miR-199a, glioma, K-RAS, chemoresistance, tumorigenesis

## Abstract

Glioma is the most malignant brain tumors in the world, the function and molecular mechanism of microRNA-199a (miR-199a) in glioma is not fully understood. Our research aims to investigate miR-199a/K-RAS axis in regulation of glioma tumor growth and chemoresistance. The function of miR-199a in glioma was investigated through *in vitro* and *in vivo* assays. We found that miR-199a in tumor tissues of glioma patients was significantly downregulated in this study. Kinase suppressor of ras 1 (K-RAS), was indicated as a direct target of miR-199a, as well as expression levels of K-RAS were inversely correlated with expression levels of miR-199a in human glioma specimens. Forced expression of miR-199a suppressed AKT and ERK activation, decreased HIF-1α and VEGF expression, inhibited cell proliferation and cell migration, forced expression of K-RAS restored the inhibitory effect of miR-199a on cell proliferation and cell migration. Moreover, miR-199a renders tumor cells more sensitive to temozolomide (TMZ) via targeting K-RAS. *In vivo* experiment validated that miR-199a functioned as a tumor suppressor, inhibited tumor growth by targeting K-RAS and suppressed activation of AKT, ERK and HIF-1α expression. Taken together, these findings indicated that miR-199a inhibits tumor growth and chemoresistance by regulating K-RAS, and the miR-199a/K-RAS axis is a potential therapeutic target for clinical intervention in glioma.

## Introduction

Malignant gliomas, as most common brain tumors around the world (1, 2), are classified according to their degree of malignancy as Grades I to IV (3, 4). Glioma clinical treatment includes surgery, chemotherapy and radiotherapy (5, 6). The most malignant grade IV glioma, glioblastomamultiforme (GBM), has an average life expectancy of only 15 months (7). Investigation of glioma carcinogenesis mechanisms would improve clinical diagnosis, drug therapy and prevention of glioma.

MicroRNAs (miRNAs) are a class of endogenous 18–22 nucleotides RNA molecules (8, 9), which always bind to the 3′-untranslated region (UTR) of specific target mRNAs, and then regulate expression of several genes at the post-transcriptional level (10–14). Accumulated evidence has clearly demonstrated that aberrant miRNA expression profiles (15) and dysregulations of specific miRNAs and their target genes, are closely associated with tumor initiation and promotion in glioma (16, 17). In particular, the miR-199a has demonstrated to suppress tumor growth in a variety of cancers including esophageal, liver, and colorectal cancers. So far, the reported miR-199a downstream target genes include oncogenes PHLPP1, E2F3, FZD6/7, HK2, and MAP3K11 (18–23), which are functioned in pathogenesis of various cancers.

K-RAS, which is reported as a family member of Ras oncogene, has involved in regulation of some cellular signal transductions (24). K-RAS is implicated in the pathogenesis of various tumors, such as GBM and pilocytic astrocytoma (25, 26). Activation of K-RAS could promote the activations of several downstream molecules, such as MAPK and ERK to regulate biological processes (27, 28).

In this study for the first time in glioma, we demonstrated the loss of miR-199a, and that K-RAS is an important direct target gene of miR-199a. Results from *in vitro* studies in human glioma U87 and U251 cells indicated that forced expression of miR-199a downregulated K-RAS signaling and suppressed cancer development and Temozolomide (TMZ) chemoresistance. The forced miR-199a overexpression also inhibited AKT and ERK1/2 pathways, through K-RAS signaling. The *in vivo* studies further demonstrated that over-express of miR-199a exhibited reduced tumor growth with down-regulated K-RAS/AKT/ERK/HIF-1α signalings. These results suggested that the loss of miR-199a/K-RAS signaling in glioma plays a pivotal role in glioma progression, and it is a potential novel targets for future clinical treatment.

## Materials and Methods

### Specimen Collection

Human glioma specimens (*n* = 24) and normal brain tissues (*n* = 9) were collected from patients in Nanjing University Medical School, China. Samples were obtained from patients with informed consents and were histologically classified by clinical pathologist.

### Cell Culture and Reagents

Human glioma cells (U87, U251) were cultured in DMEM medium. Antibodies against anti-GAPDH and anti-HIF-1α were purchased from Bioworld Technology (Atlanta, USA). Antibodies against anti-p-AKT (Ser473), anti-AKT, anti-p-ERK1/2 and anti-ERK1/2 were purchased from Cell Signaling Technology (Danvers, USA), and antibody against K-RAS was purchased from Santa Cruz (Santa Cruz, USA). TMZ (Sigma-Aldrich, USA) was used for *in vitro* chemosensitivity assay.

### Real-Time PCR

RNAs were isolated from human specimens and cells using Trizol (Invitrogen, USA). To measure expression levels of miR-199a (U6 as internal control) and mRNA levels of K-RAS(GAPDH as internal control), the cDNAs were amplified by Real-time PCR with SYBR Green reagents (Vazyme, China) on a 7900HT system(Applied Biosystems), and fold changes were analyzed by relative quantification (2^−ΔΔ*Ct*^).

Primers for K-RAS and GAPDH as below:

K-RAS: Forward Primer: ACAGAGAGTGGAGGATGCTTT, Reverse Primer: TTTCACACAGCCAGGAGTCTT; GAPDH: Forward Primer: ACAACTTTGGTATCGTGGAAGG, Reverse Primer: GCCATCACGCCACAGTTTC.

### Immunoblotting

According to the manufacturer's instruction, cell lysates in this study were prepared using RIPA buffer and indicated protease inhibitors. Aliquots of protein lysates from treated cells were fractionated by SDS-PAGE, after electrophoresis, transferred to a PVDF membrane (Roche, Switzerland), and then subjected to immunoblotting analysis.

### Cell Proliferation Assay

Indicated miR-NC and miR-199a stable cell lines were plated for 2 × 10^3^ cells per well. To evaluate the proliferation activity of miR-199a in glioma cells, according to the manufacturer's instruction, cell proliferation rate was analyzed with CCK-8 kit (Dojindo Laboratories, Japan).

### Migration Assay

Migration assay was conducted with migration chambers (BD Biosciences, UK). 5 × 10^4^ cells were plated per well in the upper chamber without serum, and the lower chamber was filled with DMEM medium with 10% FBS. 18–20 h later, the bottom cells were fixed and stained, while non-invading cells were removed. Finally, cells were extracted by 33% acetic acid and quantitatively detected (OD at 570 nm).

### Luciferase Reporter Assay

3′-UTR region of K-RAS containing software predicted miR-199a-matching seed sites (WT) and corresponding mutant sites (mut) were amplified by High fidelity PCR, and inserted into pMIR-REPORTER vector (Ambion, USA). Dual-luciferase activities were analyzed in U87 cells by the Reporter Assay (Promega, USA).

### Apoptosis Assay

Apoptosis assay (BD Pharmingen) in indicated cells were conducted according to the manufacturer's instruction with AnnexinV staining. Then samples were analyzed by flow cytometry (FACS Canto II, BD Biosciences). These data were analyzed by FlowJo software.

### *In vivo* Tumor Growth Assay

Nude mice (4-weeks-old) were purchased from Animal Center (Shanghai, China), and then bred in special pathogen-free condition. Cells (5 × 10^6^) were then injected subcutaneously into the posterior flanks of each nude mouse. Tumor sizes were measured by vernier caliper using the formula, that is volume = 0.5 × (Length × Width^2^). Twenty-four days later, mice were sacrificed as well as tumors were dissected. All mice used in this study were sacrificed according to the institutional guidelines and regulations.

### Statistical Analysis

All cellular experiments were performed three times. Data were analyzed with GraphPad Prism 5 software. The correlations between miR-199a and K-RAS in human glioma tissues were analyzed by Pearson's test. The differences were considered as statistically significant at *P* < 0.05 by *t*-test.

## Results

### Loss of MiR-199a in Human Glioma Specimens

**Figure 1:**
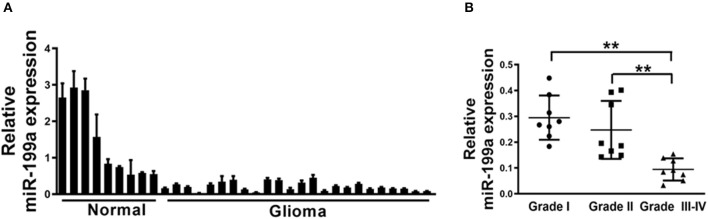
Loss of MiR-199a in human glioma specimens. **(A)** Relative miR-199a expression levels were analyzed by Real-time RT-PCR in glioma specimens (*n* = 24) and adjacent normal tissues (*n* = 9). U6 RNA levels were used as an internal control; **(B)** Relative expression levels of miR-199a in cancer tissues at Grades I, II and III-IV (for each grade, *n* = 8). Data represent mean ± SD. from three replicates. **Indicates significant difference at *p* < 0.01 when compared Grade I or Grade II group with Grade III–IV group.

Since mechanism of miR-199a is not fully understood in glioma, qRT-PCR analysis was then performed to determine indicated expression levels of miR-199a in human glioma specimens. The results clarified that miR-199a expression in tumor (*n* = 24) tissues were significantly lower, as compared to normal (*n* = 9) tissues (Figure 1A). Furthermore, in tumor tissues of glioma patients, we showed that miR-199a expression were correlated with the clinical stages, which indicated that miR-199a in high grade tumors (*n* = 8, WHO Grades III-IV) were significantly lower when compared to those in low grade tumors (*n* = 8, WHO Grade I and *n* = 8, WHO Grade II) (Figure 1B). Thus, our results indicated that miR-199a may be a potential novel biomarker for glioma staging.

### Forced Overexpression of miR-199a Inhibited Cell Proliferation and Migration Activity in Human Glioma U87 and U251 Cells

**Figure 2:**
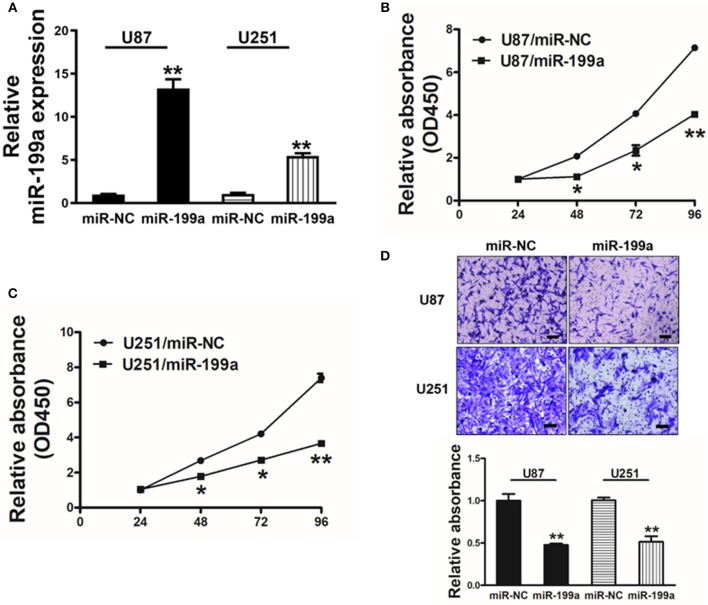
Forced overexpression of miR-199a inhibited cell proliferation and migration in human glioma U87 and U251 cells. **(A)** Relative expression levels of miR-199a in U87 and U251 stable cell lines were determined by real-time RT-PCR; **(B,C)** Cells were plated with 2,000 cells per well in 96-well plates, and cell proliferation was determined using Cell Counting Kit-8 (CCK-8) by detecting the absorbance at 450 nm at indicated time points; **(D)** Migration assay of cells were performed as previously described. Data represent mean ± SD. from three replicates. *Indicates significant difference at *p* < 0.05 when compared to miR-NC group; **Indicates significant difference at *p* < 0.01 when compared to miR-NC group.

To overexpress miR-199a, human glioma cells U87 and U251 were infected with lentivirus expressing miR-199a, and lentivirus expressing miR-NC was used as control. Stable cell lines which termed as U87/miR-NC, U87/miR-199a, U251/miR-NC, and U251/miR-199a were established after puromycin selection, and higher miR-199a expression in U87/miR-199a and U251/miR-199a were demonstrated by qRT-PCR (Figure 2A). Overexpression of miR-199a markedly attenuated cell proliferation activity in U87/miR-199a (Figure 2B) and U251/miR-199a cells (Figure 2C). In addition, forced expression of miR-199a markedly reduced cell migration activity (Figure 2D). These results are consistent with the tumor suppressor activities of miR-199a in glioma cells.

### K-RAS Is a Direct Target of miR-199a

**Figure 3:**
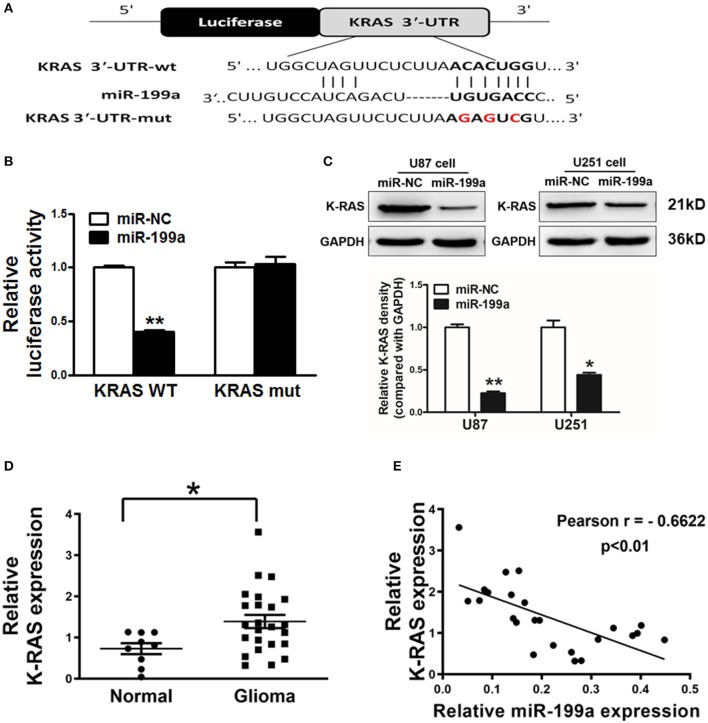
K-RAS is a direct target of miR-199a, and there is a inverse correlation between the expression levels of K-RAS and miR-199a in tumor tissues. **(A)** The complementary pairings of miR-199a with K-RAS wild-type (WT) and mutant (mut) 3′-UTR reporter constructs are shown. The mutant nucleotides of the K-RAS 3′-UTR region were labeled in red; **(B)** U87 cells were co-transfected with the reporter constructs containing the WT or mut K-RAS 3′-UTR, miR-NC or miR-199a and pRL-TK plasmids. The luciferase activities were analyzed 24 h after the transfection; **(C)** U87 cells were transfected with miR-199a or miR-NC as above. After 72 h, the expression levels of K-RAS and GAPDH in the cells were determined using immunoblotting assay; **(D)** The expression levels of K-RAS in normal tissues and human glioma specimens were determined by RT-qPCR analysis, and fold changes were obtained by the ratios of K-RAS to GAPDH levels; **(E)** Pearson′s correlation analysis was used to determine the correlation between expression levels of K-RAS and miR-199a in human glioma specimens. These data are means ± SD. from separate experiments. *Indicates significant difference at *p* < 0.05; **Indicates significant difference at *p* < 0.01.

TargetScan software was used to predict the direct targets of miR-199a, and K-RAS was found to be a potential target (Figure 3A). We constructed luciferase reporter plasmids with either the putative K-RAS wild-type binding sites (WT) or seed sequence mutant sites (mut). Luciferase assay were used to investigate whether the K-RAS is a candidate target of miR-199a. Our results clarified that overexpressing miR-199a in U87 cells reduced the luciferase activity of WT K-RAS reporter by 65%, whereas it did not change the mutant luciferase activities (Figure 3B). Furthermore, forced overexpression of miR-199a significantly attenuated protein expression of K-RAS in U87 and U251 cells (Figure 3C), suggesting that in human glioma cells, miR-199a targets K-RAS directly by binding with its 3′-UTR.

### Inverse Correlations of Lower miRNA-199a and Higher K-RAS Levels in Human Glioma Patient Tissues

To further support the notion that K-RAS oncogene is a direct target, we tested expression levels of K-RAS in human glioma specimens. Our results clarified that K-RAS expression levels were significantly higher in tumor tissues compared with normal tissues (Figure 3D). Furthermore, we determined the correlation between miR-199a and K-RAS oncogene levels in human glioma specimens using Pearson's correlation analysis. Inverse correlation in Figure 3E was found between K-RAS and miR-199a in the human glioma specimens (Pearson's correlation, *r* = −0.6622).

### Forced Expression of miR-199a Reduced Activation of K-RAS Downstream Molecules AKT and ERK1/2 as Well as HIF-1α and VEGF Expression Levels in U87 Cells

**Figure 4:**
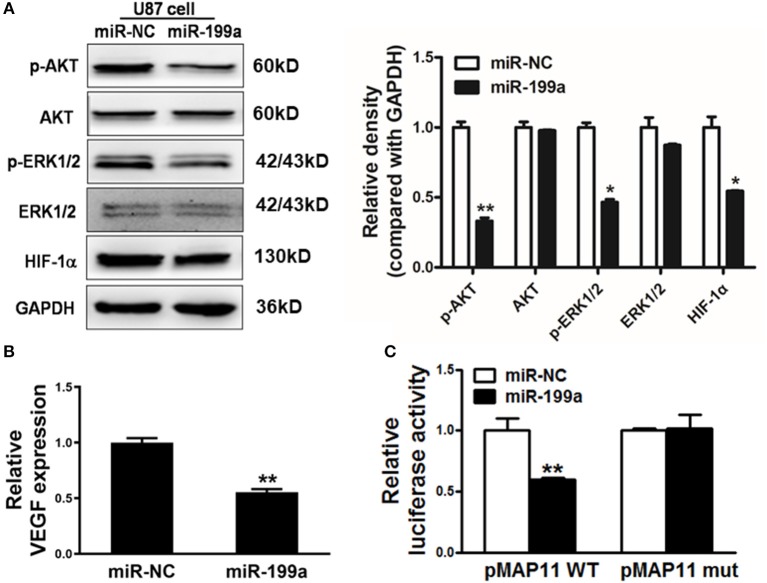
MiR-199a inhibited AKT and ERK1/2 pathways as well as the expression of HIF-1α and VEGF. **(A)** The expression levels of phosphorylated AKT (p-AKT), phosphorylated ERK1/2 (p-ERK1/2), total AKT, total ERK1/2 and HIF-1α in cells were detected by immunoblotting assay; **(B)** VEGF levels were measured by RT-qPCR in stable cell lines overexpressing miR-NC or miR-199a and normalized to level of GAPDH. *Indicates significant difference at *p* < 0.05 when compared to miR-NC group; **(C)** The wild type VEGF reporter plasmid pMAP11-WT or mutant reporter plasmid pMAP11-mut was co-transfected with pRL-TK plasmid and miR-NC or miR-199a. Firefly and Renilla luciferase activities were measured 24 h later. Data represent mean ± SD. of three replicates. **Indicates significant difference at *p* < 0.01 when compared to miR-NC group.

The downstream molecules of RAS signaling are AKT and ERK1/2, which are linked to effectors, such as hypoxia-inducible factor 1α (HIF-1α) as well as vascular endothelial growth factor (VEGF). We found that overexpression of miR-199a in U87 cells dramatically suppressed AKT and ERK1/2 activation as indicated by significantly reduced phosphorylated AKT and ERK1/2 levels and a reduction of HIF-1α protein level without change of total protein levels (Figure 4A).

Accumulated research have shown the importance role of HIF-1α/VEGF in the regulation of glioma angiogenesis (29, 30). Our previous study has reported that HIF-1α promotes VEGF gene expression through binding to the hypoxia response element (HRE) in the promoter region of VEGF. In addition to decreased HIF-1α, we also detected a significant reduction of VEGF mRNA levels in miR-199a-expressing U87 cells (Figure 4B). To test whether miR-199a inhibited VEGF expression through HIF-1α, we analyzed and compared effects of miR-199a on (1) a VEGF promoter reporter plasmid (pMAP11WT) containing the HIF-1α binding site, and (2) a mutant (pMAP11 mut) plasmid with mutation, overexpression of miR-199a inhibited the luciferase activities of the VEGF promoter reporter plasmid as shown in Figure 4C. Thus, our results indicated that miR-199a suppressed the gene expression of VEGF through HIF-1α.

### Restoration of K-RAS Reversed miR-199a Mediated Suppression on Cell Proliferation and Migration

**Figure 5:**
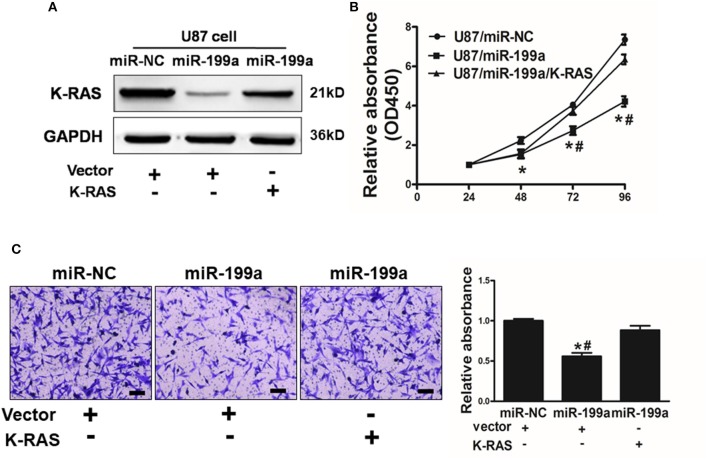
Overexpression of K-RAS cDNA reversed miR-199a-suppressed cell proliferation and cell migration. **(A)** U87 cells were co-transfected with miR-199a precursor or miR-NC, and empty vector or K-RAS cDNA without 3′-UTR. After 72 h, immunoblotting assay was performed as described above; **(B)** Cells were treated as above, and cell proliferation assay was determined using CCK-8 kit; **(C)** U87 cells overexpressing miR-NC or miR-199a were treated as described above, cell migration was determined using 24-well BD migration chambers and quantified using a standard microplate reader (OD at 570 nm). Data represent mean ± SD. of three replicates. *Indicates significant difference at *p* < 0.05 compared to miR-NC control; ^#^Indicates significant difference at *p* < 0.05 compared to miR-199a and K-RAS overexpression group.

To further demonstrate the role of K-RAS in miR-199a mediated effects on cell proliferation and migration, U87/miR-NC cells or U87/miR-199a cells were co-transfected with control vector (Vector) or K-RAS without 3′-UTR. miR-199a reduced K-RAS expression significantly, and forced expression of K-RAS restored K-RAS expression as shown in Figure 5A. We also determined the effect on cell proliferation activity. As shown in Figure 5B, U87/miR-199a cells has significantly reduced cell proliferation rate, while overexpressing K-RAS in U87/miR-199a cells (U87/miR-199a/K-RAS), restored the cell proliferation rate. The cell migration was determined and quantified by a microplate reader using migration chambers (OD at 570 nm). As we expected, overexpression of K-RAS (U87/miR-199a/K-RAS) restored miR-199a-inhibited cell migration activity (Figure 5C). To sum up, these results suggested that miR-199a suppresses human glioma cell proliferation and migration, and forced expression of K-RAS reversed miR-199a effects.

### Overexpression of miR-199a Rendered Cells More Sensitive to TMZ Through Targeting K-RAS

**Figure 6:**
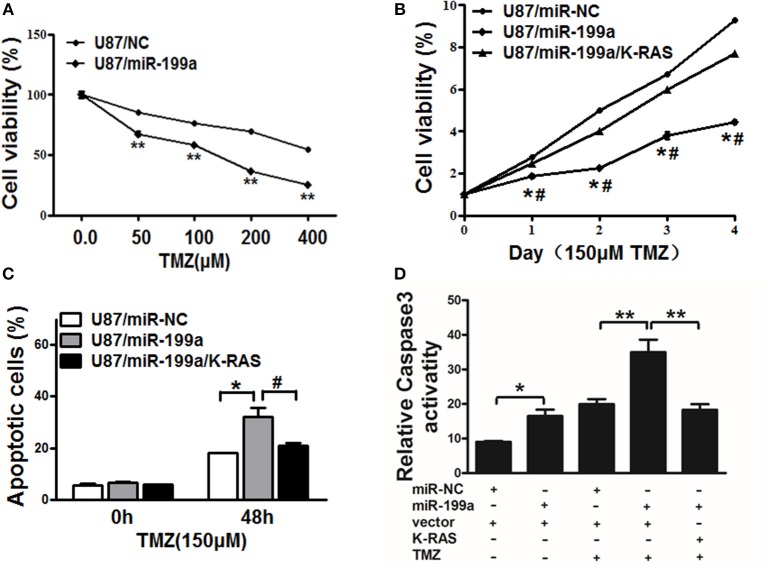
MiR-199a increased TMZ chemosensitivity by targeting K-RAS in glioma cells. **(A)** U87 cells stably expressing miR-NC or miR-199a were treated with different concentrations of TMZ for 48 h, and cell viability was analyzed using CCK-8 assay; **(B)** U87 cells stably expressing miR-NC, miR-199a, or miR-199a in combination with K-RAS overexpression were treated with 150 μM of TMZ for indicated time points. Cell viability was analyzed by CCK-8 assay; **(C,D)** Cell apoptosis was analyzed by flow cytometry and by caspase-3 assay. Data represent mean ± SD. from three replicates. *Indicates significant difference at *p* < 0.05 compared to miR-NC control; **Indicates significant difference at *p* < 0.01 compared to miR-NC control; ^#^Indicates significant difference at *p* < 0.05 compared to miR-199a and K-RAS overexpression group.

TMZ chemoresistance is the major obstacle in process of glioma chemotherapy. In this study, U87/miR-NC cells or U87/miR-199a were treated with TMZ at different concentrations for 2 days, as shown in Figure 6A, U87/miR-199a cells were significantly more sensitive to TMZ treatment. Furthermore, overexpressing K-RAS in U87/miR-199a cells (U87/miR-199a/K-RAS) nearly completely reversed the chemosensitivity to TMZ treatment (Figure 6B). We further investigated the role of miR-199a/K-RAS axis in TMZ mediated apoptosis by apoptosis analysis and caspase-3 activity assay. As shown in Figure 6C, TMZ treatment produced significantly higher apoptotic cell population in U87/miR-199a cells as compare to the control U87/miR-NC cells. Forced expression of K-RAS in U87/miR-199a cells (U87/miR-199a/K-RAS) nearly completely reversed the effect. Moreover, activities of caspase-3 were determined. As shown in Figure 6D, compared to the negative control (Bar 1, U87/miR-NC), overexpression of miR-199a (Bar 2, U87/miR-199a) significantly increased caspase 3 activity. TMZ treatmentof control U87/miR-NC cells increased caspase3 activity (Bar 3, U87/miR-NC + TMZ), and TMZ treatment of U87/miR-199a cells strongly and significantly increased caspase3 activity (Bar 4, U87/miR-199a + TMZ). In addition, overexpression of K-RAS in U87/miR-199a cells attenuated the activation of caspase-3 (Bar 5, U87/miR-199a/K-RAS + TMZ). To sum up, our results showed that miR-199a rendered glioma cells more sensitive to TMZ through targeting K-RAS.

### MiR-199a Suppressed Tumor Growth *in vivo*

**Figure 7:**
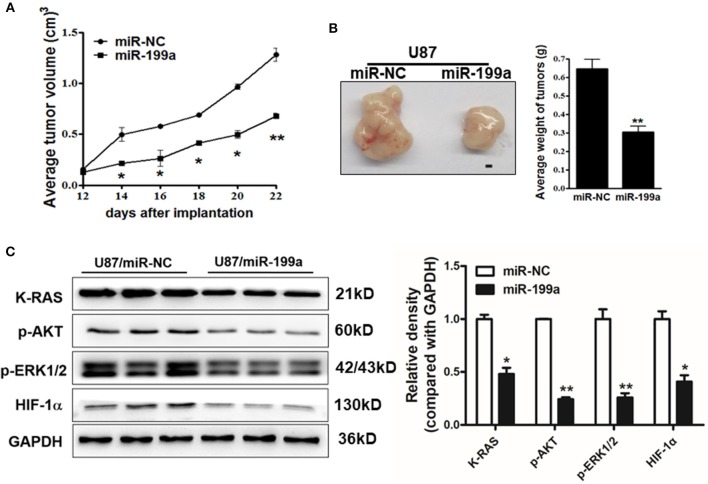
MiR-199a overexpression suppressed tumor growth and decreased expression levels of K-RAS and its downstream molecules in tumor tissues. **(A)** U87/miR-199a or U87/miR-NC cells (5 × 10^6^ cells) were dispersed in 100 μl of serum-free DMEM medium, and subcutaneously injected into posterior flanks of the nude mice (*n* = 5). Tumors were measured every 2 days after they were apparently detectable on Day 12. Tumor volumes were calculated using the following formula: volume = 0.5 × (length × width^2^); **(B)** Mice were sacrificed 24 days after implantation, tumors were harvested and photographed. Representative pictures from each group were shown (Bar = 1 mm) and tumors were weighted; **(C)** Total proteins were extracted from xenografts and subjected to immunoblotting assay to test K-RAS, p-AKT, p-ERK1/2, HIF-1α, and GAPDH expression levels. Data were presented mean ± SD. *Indicates significant difference at *p* < 0.05 when compared to miR-NC group; **Indicates significant difference at *p* < 0.01 when compared to miR-NC group.

To determine whether over-expression of miR-199a inhibited tumor growth *in vivo*, U87/miR-NC cells and U87/miR-199a cells were injected into immunodeficient nude mice. After cell injection, tumor sizes were measured. Compared to control mice injected with U87/miR-NC cells, mice injected with U87/miR-199a cells developed significantly smaller tumors from Day 14 (Figure 7A). Twenty-four days later, Mice were sacrificed after implantation, tumors were harvested, photographed, and weighed. A pair of representative tumors trimmed out from U87/miR-199a and U87/miR-NC groups, and the average tumor weight were shown in Figure 7B. Forced expression of miR-199a produced tumors with significantly lower tumor weight. We also determined the protein levels of K-RAS, p-AKT, p-ERK1/2 and HIF-1α in tumor tissues, and found that these proteins from U87/miR-199a group were significantly lower than that of U87/miR-NC group, which is consistent with *in vitro* data (Figure 7C). Our results suggested that miR-199a inhibited tumor growth by inhibiting K-RAS expression and its downstream molecules.

## Discussion

MicroRNAs (miRNAs) in diverse human cancers have been frequently indicated to be dysregulated (31, 32). The miR-199a have reported to be downregulated in multiple malignancies (33–35). We first demonstrated that expression levels of miR-199a was downregulated in clinical glioma samples, and function as a tumor suppressor to increase sensitivity to treatment.

The reported miR-199a targets include GRP78, GSK-3β, Discoidin domain receptor 1 (DDR1), mTOR, CD44, and IκB kinase-beta. It works through targeting GRP78, a major endoplasmic reticulum chaperone, in prostate cancer cells to induce apoptosis and increase sensitivity to trichostatin A, the histone deacetylase inhibitor (36); through GSK-3β in renal cell cancer cells to decreases cell proliferation (37); through a receptor tyrosine kinase DDR1, to suppress invasiveness and migratory ability of colorectal cancer cells (38); through the mTOR and CD44 to increase sensitivity to cisplatin treatment and to reduce the number of ovarian cancer stem cells (39, 40). Finally, it works through targeting IκB kinase-beta to increase TNF-α-induced ovarian cancer cell apoptosis (41). Here, we first identified K-RAS as a novel target of miR-199a. We also confirm the inverse correlation between miR-199a and K-RAS levels in glioma specimens.

Temozolomide (TMZ) is a first-line drug for glioma treatment. A recent study has shown that miR-29c contributed to sensitize cells to temozolomide treatment by targeting O^6^-methylguanine-DNA methyltransferases in glioma (42). On the other hand, miR-423-5p was reported to function as a oncogene and promoted chemoresistance to temozolomide in glioblastomas (43). Here we found that overexpression of miR-199a rendered cells more sensitive to TMZ through its target K-RAS. Thus, miR-199a/K-RAS signaling may be a potential new target to overcome chemoresistance to TMZ in glioma.

## Conclusions

To sum up, we have clarified that K-RAS is a novel direct target of miR-199a. MiR-199a inhibit activity of cell proliferation, cell migration, drug chemoresistance and tumor growth by regulating K-RAS via AKT and ERK signalings. These results elucidated that miR-199a/K-RAS in the future may be used as a target for glioma treatment.

## Data Availability Statement

The datasets generated for this study are available on request to the corresponding author.

## Ethics Statement

The studies involving human participants were reviewed and approved by Ethics Committee of Nanjing University. The patients/participants provided their written informed consent to participate in this study. The animal study was reviewed and approved by Ethics Committee of Nanjing University.

## Author Contributions

WL, LW, and XG carried out the samples collection and performed the experiments. X-BJ and L-HW revised the manuscript. W-TL, LC, ZZ, Z-MS, L-ZL, and ML designed the studies. WL, LW, J-YC, and B-HJ wrote the manuscript.

### Conflict of Interest

The authors declare that the research was conducted in the absence of any commercial or financial relationships that could be construed as a potential conflict of interest.
